# Cecropin A Alleviates Inflammation Through Modulating the Gut Microbiota of C57BL/6 Mice With DSS-Induced IBD

**DOI:** 10.3389/fmicb.2019.01595

**Published:** 2019-07-10

**Authors:** Zhenya Zhai, Fan Zhang, Ruihua Cao, Xiaojun Ni, Zhongquan Xin, Jinping Deng, Guoyao Wu, Wenkai Ren, Yulong Yin, Baichuan Deng

**Affiliations:** ^1^Guangdong Provincial Key Laboratory of Animal Nutrition Control, National Engineering Research Center for Breeding Swine Industry, College of Animal Science, Subtropical Institute of Animal Nutrition and Feed, South China Agricultural University, Guangzhou, China; ^2^Department of Animal Science, Texas A&M University, College Station, TX, United States; ^3^National Engineering Laboratory for Pollution Control and Waste Utilization in Livestock and Poultry Production, Key Laboratory of Agro-ecological Processes in Subtropical Region, Institute of Subtropical Agriculture, Chinese Academy of Sciences, Changsha, China

**Keywords:** inflammatory bowel disease, antimicrobial peptide, gut microbiota, intestinal barrier, C57BL/6 mice

## Abstract

The present study is undertaken to assess the alleviating effects of antimicrobial peptide cecropin A on inflammatory bowel disease (IBD) in C57BL/6 mice and changes in the gut microbiota, compared to an antibiotic gentamicin. Different doses of cecropin A were intraperitoneally injected into C57BL/6 mice for 5 days to determine the safe doses. The injection doses at ≤ 15 mg/kg showed no negative impact on the liver, heart, spleen, and kidney. The severe and moderate IBD mice model was successfully established via supplementation of 4 or 2.5% dextran sulfate sodium (DSS) in drinking water for 5 days. The severe IBD model was used to ensure the optimal therapeutic dose of cecropin A. Survival rate, body weight and disease activity index (DAI) scores were measured. Administration of 15 mg/kg, not 5 mg/kg cecropin A, for 5 days increased survival rate and decreased body weight loss of mice. The moderate IBD model was applied to investigate the mechanisms for cecropin A to alleviate inflammation in comparison to gentamicin. The mice were treated with 15 mg/kg cecropin A or 5 mg/kg gentamicin for 3 days. The levels of cytokines and related proteins in the colon were detected by ELISA and Western blotting. The microbiota in cecum contents were analyzed using 16S rRNA gene sequencing. The results showed that cecropin A and gentamicin relieved body weight loss, DAI, and gut mucosa disruption, while decreasing tumor necrosis factor-α (TNF-α), interlukin-1β (IL-1β), and interlukin-6 (IL-6) induced by DSS. In addition, cecropin A and gentamicin showed different effects on the gut microbiota structure. Both cecropin A and gentamicin decreased DSS-induced enrichment of *Bacteroidaceae* and *Enterobacteriaceae*. However, cecropin A showed a selective enrichment of *Lactobacillus* in contrast to gentamicin, which demonstrated a selective effect on *Desulfovibrionaceae* and *Ruminococcaceae*. Cecropin A alleviates IBD through decreasing harmful gut microflora and specifically enhancing beneficial gut microflora. The mechanism of this effect is different from gentamicin.

## Introduction

Inflammatory bowel disease (IBD), including Crohn’s disease (CD), and ulcerative colitis (UC), is a category of gastrointestinal disease, which is characterized by bloody diarrhea, abdominal pain, systemic infection, and even death (Constante et al., 2017). New epidemiological studies show that more than 2 million people in western countries suffer from IBD, and the incidence is developing rapidly (Ng et al., 2018). Although the exact pathogenesis remains unclear, intestinal flora disorder is the one of the most key factors (He et al., 2012), which may lead to the mucosal dysfunction (Pineton et al., 2010) and immune dysregulation (Yadav et al., 2016). The appropriate composition of the intestinal microbiota is essential for hosts to maintain gastrointestinal tract health (Mottawea et al., 2016). Several kinds of “harmful flora,” such as *Escherichia coli (E⋅coli)*, *Enterobacteriaceae*, and *Bacteroides*, are considered as biomarkers in IBD (Berry and Reinisch, 2013). The pathogenic bacteria such as *E⋅coli* could destroy intestinal barrier and invade the body (Bucker et al., 2014). In contrary to the harmful flora, several probiotics and their metabolites, may suppress the proliferation of pathogenic bacteria (Isaacs and Herfarth, 2008; Bian et al., 2016), increase the barrier function (Miyauchi et al., 2009), and relieve enteritis (Hu et al., 2018).

Antibiotics such as vancomycin, gentamicin and azithromycin are proven to be effective drugs to cure IBD through elimination of harmful bacteria and reducing inflammation (Khan et al., 2011). However, some of the pathogenetic bacteria, especially gram-negative bacteria, such as *Salmonella enteritidis* may obtain drug resistance and weaken the therapy effect (Meakins et al., 2008; Jess et al., 2011). Antibiotics supplementation to feed for enteritis prevention or treatment in pigs or other animals (Rhouma et al., 2016) lead to antibiotic residues in livestock products (Darwish et al., 2013). In addition, exposure to antibiotics also induces the imbalance of gut microbiota (Kronman et al., 2012; Ledder and Turner, 2018). Finding effective alternatives to antibiotics has become an increasingly urgent task both in medicine or animal husbandry.

Among the potential alternatives, antimicrobial peptides (AMPs) are particularly important, due to their broad-spectrum antimicrobial activity and low risk to cause bacterial resistance. Exogenous antimicrobial peptides play important roles in alleviate enteritis. Dietary supplement with cecropin AD may relieve enterotoxigenic *Escherichia coli-*induced piglet diarrhea (Wu et al., 2012). Intraperitoneal injection or rectal administration of cathelicidin-WA also effectively remits lipopolysaccharide (LPS)- or DSS-induced enteritis in mice (Zhang et al., 2015; Yi et al., 2016, 2017). Cecropin A is one of the earliest cecropins discovered from *Hyalophora cecropia* and has high bacteriostatic activity to gram-negative bacteria (Steiner et al., 1981). In the past decades since cecropin A was found, the antibacterial mechanisms of cecropin A have been well researched (Rangarajan et al., 2013). Besides, cecropin A is commonly used as a template for peptide molecular hybrids (such as melittin) to enhance the antibacterial activity of AMPs and reduced the cell toxicity (Shin et al., 1999; Rai et al., 2016). In addition, our previous studies also showed that cecropin A could increase intestinal barrier function on IPEC-J2 cell model (Zhai et al., 2018). However, whether cecropin A could alleviate enteritis and regulate gut microbiota *in vivo* is unknown.

In this study, we assumed that cecropin A has a therapeutic effect on IBD through regulating gut microbiota. Cecropin A and gentamicin were used to treat DSS-induced IBD. The changes in the gut microbiota were measured by using 16s rRNA gene sequencing. This study provides a direct comparison on the modulations of gut microbiota structure by cecropin A and gentamicin, which may help to shed light on the different mechanisms for the actions of AMPs or antibiotics.

## Materials and Methods

### Ethics Statement

The experimental design and procedures in this study were reviewed and approved by the Animal Care and Use Committee of the Institute of Subtropical Agriculture, Chinese Academy of Sciences (No. ISA-2018-035).

### Peptide Synthesis

The cecropin A were synthesized and purified by a peptide company (DgPeptides Co., Ltd., Hangzhou, China), and the sequences were confirmed via matrix-assisted laser desorption/ionization time-of-flight mass spectrometry (MALDI-TOF MS). The purity of cecropin A was higher than 95%, which was measured by reversed-phase high-performance liquid chromatography.

### Animals

C57BL/6 mice (3 weeks of age) were purchased from Guangdong Medical Laboratory Animal Center. The mice were housed for 3 per cage under the same condition (temperature, 24 ± 1^∘^C; lighting cycle, 12 h:12 h light/dark; 7:00–19:00 for light) and had free access to food and drinking water.

#### Establishment of 4% DSS Induced IBD Model in Mice and Treatment With Different Dose of Cecropin A

**FIGURE 1:**
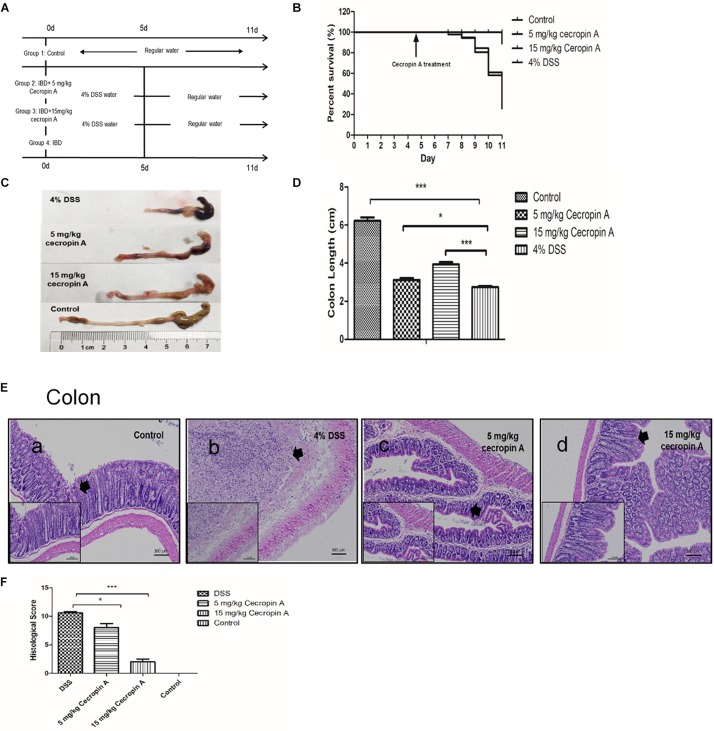
The therapeutic effect of intraperitoneal injection of cecropin A on survival rate, colon length, and colon epithelium mucosa recovery in DSS-treated mice. **(A)** the experimental design; **(B)** survival rates of mice; **(C,D)** colon length; **(E)** colon morphology; **(F)** the histological score. The survival rate was measured by using the Kaplan–Meier method and the body weight change, diarrhea score and DAI were analyzed by one-way ANOVA. The data are shown as mean ± SEM with, ^*^P < 0.05, ^∗∗∗^*P* < 0.001. Because the survival rate changed every day, except for the control group (*n* = 9), the number of mice was changed in the 5 mg/kg, 15 mg/kg cecropin A or 4% DSS groups.

The experimental design is shown in Figure 1A. Mice were housed until 6–7 weeks of age, and then divided into four groups (*n* = 9 per group). The IBD model was induced by giving water containing 4% (weight: volume) DSS (36,000–50,000 M. Wt., MP biomedicals, Solon, OH, United States) for 5 days (D0–D5). Then the mice were intraperitoneally injected with saline, 5 mg/kg cecropin A or 15 mg/kg cecropin A for 5 days (D5–D10). The mice in the control group received drinking water and were intraperitoneally injected with saline. All the mice were weighed every day. The survival rate, disease activity index (DAI) was calculated by a well-established method (De Fazio et al., 2016; Zhou et al., 2018) and validated scores are showed in Supplementary Table S1. After treatment for 5 days, the mice were sacrificed by CO_2_ (D11), the liver, distal ileum (near the cecum for 2 cm) and colon were collected and stored in 4% neutral polyformaldehyde fixative for 48 h. The tissue was embedded with paraffin for further section and staining.

#### Establishment of 2.5% DSS-Induced IBD Model in Mice and Treated With Cecropin A and Gentamicin

**FIGURE 2:**
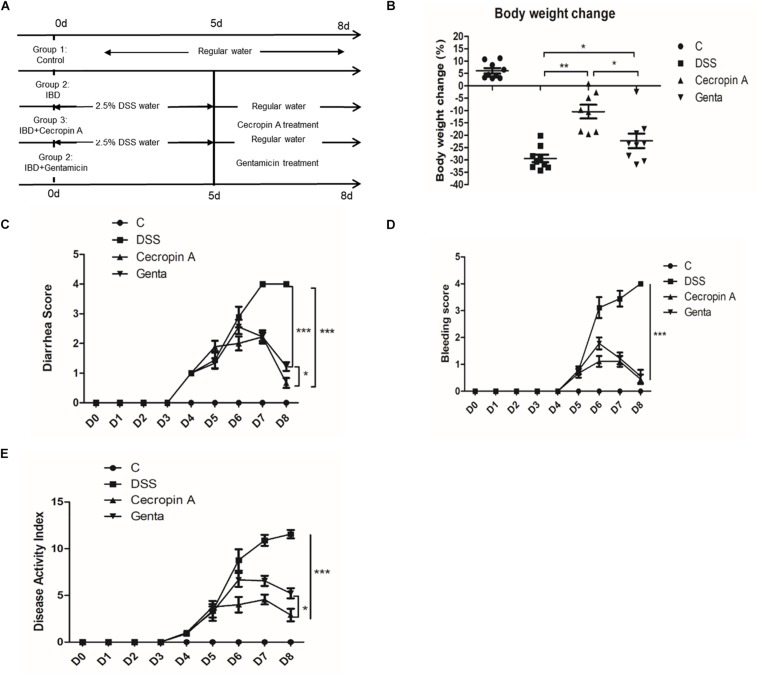
Intraperitoneal injection of cecropin A alleviated DSS-induced IBD in C57BL6 mice. **(A)** the experimental design, **(B)** body weight changes, **(C)** diarrhea scores, **(D)** bleeding scores, and **(E)** DAI scores. C: the control group; Genta: the mice were pretreated by 2.5% DSS for 5 days and then received intraperitoneal injection of 5 mg/kg body weight gentamicin. Cecropin A: the mice were pretreated by 2.5% DSS for 5 days and then received intraperitoneal injection of 15 mg/kg body weight cecropin A. DSS: the mice were treated by 2.5% DSS. The control group and DSS group received intraperitoneal injection of saline. The mice were sacrificed at 8 days. The data are mean ± SEM and analyzed by one-way ANOVA (*n* = 9). ^*^P < 0.05, ^∗∗^*P* < 0.01, and ^∗∗∗^*P* < 0.001.

The experimental design is shown in Figure 2A. Mice were housed until 6–7 week of age and the IBD model was induced by giving water containing 2.5% DSS for 5 days (D0–D5). Cecropin A and gentamicin (J&K Scientifc Ltd., Shanghai) were dissolved in saline. Then the mice were intraperitoneally injected with saline (DSS group), 15 mg/kg cecropin A (cecropin A group) or 5 mg/kg gentamicin (gentamicin group) for 3 days (D5–D8). The dose of gentamicin was selected according to the clinical medicine instructions and guides. The mice in the control group received drinking water and were intraperitoneally injected with saline. All the mice were evaluated by body weight change, diarrhea index, fecal bleeding index and DAI every day. Then the mice were sacrificed by CO_2_ at D8. The distal ileum (near the cecum to 2 cm) and colon were collected and stored in 4% neutral polyformaldehyde fixative or frozen in liquid nitrogen for ELISA or Western blotting. The cecum contents were collected in sterile centrifuge tube and frozen in liquid nitrogen, and then stored at −80^∘^C until total genomic DNA extraction. Cecum content samples were used for subsequent 16S rRNA gene sequencing.

### Tissue Histological Examination

The experiment was performed by using hematoxylin and eosin (H&E) staining. The tissue embedded in paraffin blocks was cut into 5 μm slices, deparaffinized and hydrated, then stained with H&E. The villus length and crypt depth were measured. The histologic scoring system was measured according to the well establish standard (De Fazio et al., 2016; Zhou et al., 2018), as is shown in Supplementary Table S2.

### Cytokine and Chemokine Analysis

To demonstrate the correlation between inflammatory response and disease, the cytokines and chemokines such as TNF-α, IL-1β, and IL-6, were detected by using an enzyme linked immunosorbent assay (ELISA). The colonic samples were weighed and homogenized in PBS containing a protease inhibitor cocktail (Sangon Biotech, Shanghai, China). Then the cytokines were measured according to the ELISA kit (CUSABIO, Wuhan, China). The total protein concentration was assessed according to the BCA assay kit (Thermo Fisher Scientific, Waltham, MA, United States). The cytokine and chemokine concentration were correlated by using total protein concentration.

### Western Blotting

To test the protein level of tight junction protein (ZO-1, occludin and claudin-1), the phosphorylation level of the proteins, which belongs to the downstream of mitogen-activated protein kinase (MAPK) and NF-κB signaling pathway, were tested. The tissue samples were homogenized by using Radio Immunoprecipitation Assay (RIPA) lysis buffer containing protease inhibitor and phosphatase inhibitor cocktail (Songon Biotech, Shanghai, China). The concentration of protein was determined by using the BCA protein assay kit. The tissue sample was mixed with 5 × loading buffer (Sangon Biotech, Shanghai China) and heating at 100^∘^C for 5 min. The SDS-PAGE gel kits were purchased from Sangon Biotech (Shanghai, China). The sample (50 μg protein) was loaded to each well. The detailed process was described in the previous study (Zhai et al., 2018). The primary antibodies including β-actin, p38, p-p38, c-Jun, p-c-Jun, p65, p-p65, and ZO-1, claudin-1 were purchased from Cell signaling Technology (CST, Danvers, MA, United States) and occludin was purchased from Thermo Fisher Scientific (Waltham, MA, United States).

### 16S rRNA Sequencing With Ion S5^TM^XL Sequencing

All the sequencing procedures and general data analyses were performed by a commercial company (Novogene, Beijing, China). DNA was extracted from cecum contents by using Qiangen QIAamp DNA stool Mini Kit according to the protocol for isolation of DNA. There were 8, 7, 9, and 8 samples could extract enough DNA for the next step in control, DSS, cecropin A and gentamicin group. The V3–V4 region of the bacteria 16S ribosomal RNA gene amplified by PCR, the following primers (5′–3′) were used: 341F CCTAYGGGRBGCASCAG and 806R GGACTACNNGGGTATCTAAT. The PCR system was 30 μL, including 15 μL PhusionMaster Mix (2×; New England Biolabs, Ipswich, MA, United States), 3 μL Primer (2 μM), 10 μL gDNA (1 ng/μL), 2 μL H_2_O. The reaction system is as follows: 98^∘^C 1 min; then 30 cycles (98^∘^C, 10 s; 50^∘^C, 30 s; 72^∘^C, and 30 s); 72^∘^C, 5 min. After the PCR, the amplicons were electrophoresis in 2% agarose gel and then extracted by using the gel extraction kit (GeneJET, Thermo Fisher Scientific, Waltham, MA, United States). The gene library was constructed by using the Ion Plus Fragment Library Kit 48 rxns (Thermo Fisher Scientific, Waltham, MA, United States). Then the sequencing of genes was performed on the Ion S5^TM^ XL platform to obtain the data.

The data were measured by using Cutadapt (version 1.9.1) to treat low-quality sequence reads, then sample data were separated from reads by barcode. The original data (raw reads) were obtained by truncating barcode and primer sequence. After removal of chimera sequences, the clean data was obtained. Operational taxonomy units (OTUs) were clustered with 97% identity by using Uparse (v7.0.1001) based on clean data. The venn diagram, principal coordinate analysis (PCoA), principal component analysis (PCA), and non-metric multi-dimensional scaling (NMDS) were conducted based on OTU by the using R software. OTU is annotated and divided into phylum, class, order, family, genus and species. The linear discriminant analysis (LDA) effect size (LEfSe) was used to elucidate the differences in bacterial taxa. An LDA score ≥ 4 was considered to be an important contributor to the model. The Spearman analysis was used to measure the correlation between gut microbiota and inflammatory cytokines.

### Statistical Analyses

Data are expressed as the mean ± SEM. One-way ANOVA was used to determine differences among groups by using SPSS 20.0. Plots were performed by using GraphPad Prism 5. Differences were considered statistically significant when *P* < 0.05. The survival rate was calculated by the Kaplan–Meier method and plotted by GraphPad Prism 5.

## Results

### Effects of Intraperitoneal Injection of Cecropin A on Body Weight, Organ Index and Concentration of Serum LDH, AKP, and AST in Mice

Intraperitoneal injection of cecropin A had no effect on body weight, spleen, heart and kidney index of mice (*P* > 0.05), but significantly increased the liver index in the 30 mg/kg cecropin A group (*P* < 0.01, Supplementary Figures S1A,B).

The concentrations of AST, LDH and AKP in serum are also shown in Supplementary Figures S1C–E. The data showed that cecropin A had no significant effect on these measured variables in the 5 mg/kg and 15 mg/kg groups. But in the 30 mg/kg group, the activities of AST and AKP tended to be higher (0.05 < *P* < 0.1).

### Effects of Intraperitoneal Injection of Cecropin A on the Liver, Spleen, Ileum, and Colon Histomorphology

Owing to the higher values of liver index and AST concentration in the 30 mg/kg cecropin A group, we analyzed the liver histomorphology by using H&E staining. The results showed that the liver morphology was not affected by intraperitoneal injection of either saline or low doses (5 and 15 mg/kg) of cecropin A (Supplementary Figures S2e–g). However, the hepatic morphology and structure of mice in the 30 mg/kg group were altered by cecropin A (Supplementary Figure S2h). Intraperitoneal injection 30 mg/kg cecropin A for 5 days induced slight hepatic cell swelling, cytoplasmic loosening and reticular, while the injection of 5 mg/kg and 15 mg/kg did not show the same effects. In contrast, there were no differences in the spleen among the treatment groups (Supplementary Figures S2a–d).

To determine the effects of intraperitoneal injection of cecropin A on the ileum and colon, their histological morphologies were also evaluated by using H&E staining. The data showed that, in the 5 mg/kg group, cecropin A increased the villus length and tended to increase the villus length in the 15 mg/kg group, compared to the control group (Supplementary Figure S3). In the colon, 15 mg/kg cecropin A increased the crypt depth, when compared with the control group (*P* < 0.05). We also found that intraperitoneal injection of 30 mg/kg cecropin A for 5 days had no effect on the morphology of both the ileum and the colon. The results showed that an appropriate dose of cecropin A could be beneficial for the development of the intestinal tract.

### The Therapeutic Effect of Cecropin A on DSS-Induced IBD in Mice

To evaluate the therapeutic effect of cecropin A on mice, we used a high dose of DSS to induce a severe form of the IBD. The results showed that the affected mice had grossly diarrhea and bleeding, and their survival rate was reduced to about 26% within 4 days (D7–D11). Mice treated with 15 mg/kg cecropin A exhibited an improvement in the survival rate and a decrease in the body weight loss (*P* < 0.05), but treatment with 5 mg/kg cecropin A had no effect on survival (Figure 1B). Treatment with both 5 and 15 mg/kg cecropin A reduced body weight loss, diarrhea and DAI (Supplementary Figure S4, *P* < 0.05).

After dissection, we also measured the colon length. Although treatment with 5 mg/kg cecropin A did not improve survival rates, the colon length in mice treated with 5 and 15 mg/kg cecropin A was significantly increased, compared to the 4% DSS group (*P* < 0.05). Besides, the intestinal wall was very thin after DSS treatment, there were also black blood clots mixed with cecum and colon contents. The black blood clot was decreased in the 5 mg/kg group, and no blood clot was found in the 15 mg/kg group mice (Figures 1C,D). By using H&E staining and evaluating the histological score of colons (Figures 1E,F), we found that treatment with both 5 and 15 mg/kg cecropin A decreased the colon histological score compared to the DSS group. As the thick arrows showed, in DSS group, the epithelium of the colon disappears completely and was infiltrated by inflammatory cells. In 5 mg/kg group, although the epithelial structure was severely damaged, it was significantly better than DSS group. In 15 mg/kg group, the crypt structure was significantly restored, and the histological score is significantly reduced. The results indicate that 15 mg/kg cecropin A is appropriate for the treatment of DSS induced IBD. We also found that the better ileum epithelium recovery in 15 mg/kg group than 5 mg/kg group (Supplementary Figure S5). Furthermore, in mice treated with 15 mg/kg cecropin A, the colon histological score was also decreased, compared to the 5 mg /kg group. The data suggest that cecropin A could promote the recovery of the ileum and colon mucosa from injury in IBD mice, but 15 mg cecropin A /kg body weight was the appropriate dose to cure IBD.

### The Different Therapeutic Effect of Cecropin A and Gentamicin on DSS-Induced IBD in Mice

To evaluate the differences between cecropin A and antibiotics, gentamicin, a widely used antibiotic in IBD treatment, was used as the antibiotic control. Because of the disease and low survival rate induced by 4% DSS, the IBD model was induced by using a lower dose of DSS (2.5%) for 5 days and then treated with 15 mg/kg cecropin A or 5 mg/kg gentamicin for 3 days. The results showed that cecropin A and gentamicin significantly decreased the body weight loss, diarrhea score, bleeding score and DAI score (*P* < 0.05), compared to the DSS group (Figures 2B–E). At the same time, we also noticed that the body weight loss and diarrhea score were also lower in the 15 mg/kg cecropin A group than in the gentamicin group (*P* < 0.05), but there was no significant difference in bleeding scores between the two groups of mice. The results showed that both cecropin A and gentamicin could effectively alleviate the hemorrhage, diarrhea, but cecropin A had a better effect on body weight loss.

### The Different Effect of Cecropin A and Gentamicin on Colon Length, Epithelium Histological Morphology and Tight Junction Protein Levels in IBD Mice

**FIGURE 3:**
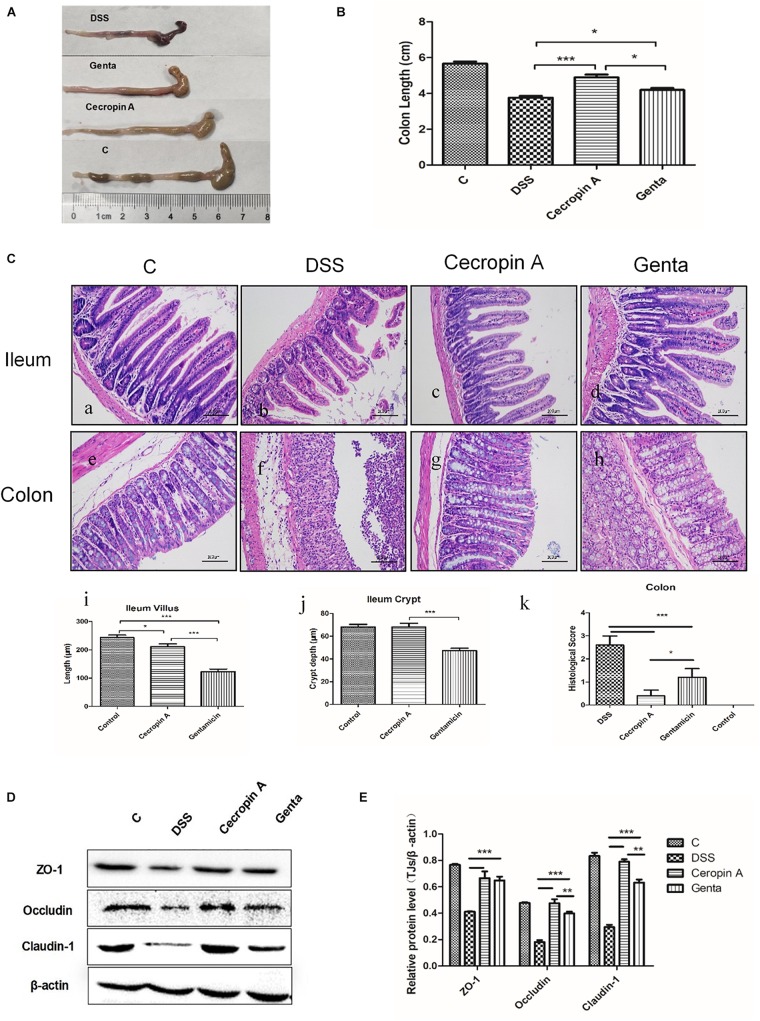
The effects of cecropin A and gentamicin on the colon length, intestinal histological morphology and TJ protein abundances in DSS-treated mice. **(A,B)** the colon length, *n* = 9; **(C)** intestinal morphology, *n* = 5; **(D,E)** TJ protein abundances, *n* = 3. C: the control group; Genta: the mice were pretreated by 2.5% DSS for 5 days and then received intraperitoneal injection of 5 mg/kg body weight gentamicin. Cecropin A: the mice were pretreated by 2.5% DSS for 5 days and then received intraperitoneal injection of 15 mg/kg body weight cecropin A. DSS: the mice were treated by 2.5% DSS. The control group and DSS group received intraperitoneal injection of saline. The data are mean ± SEM and analyzed by one-way ANOVA. ^*^P < 0.05, ^∗∗^*P* < 0.01, and ^∗∗∗^*P* < 0.001.

To evaluate the effect of cecropin A and gentamicin on the recovery of the distal ileum and colon, the colon length and histological morphology were measured. The results showed that the colon length in mice treated with cecropin A and gentamicin were increased, compared to the DSS group (Figures 3A,B; *P* < 0.05). The colon length in the cecropin A group was also significantly longer than that in the gentamicin group (*P* < 0.05). The representative image of H&E staining showed the damage and recovery in the ileum and colon mucosa (Figure 3C). The result showed that administration of 2.5% DSS induced severe mucosa damage. The villi in the ileum were disrupted while the crypt was shortened in both the ileum and the colon. In mice treated with cecropin A and gentamicin, the ileum villi and crypt showed recovery, with the crypt depth and villi length significantly being greater in the cecropin A group (*P* < 0.05) than in the gentamicin group. In the colon, histological scores in the cecropin A group were decreased, compared to the gentamicin group (*P* < 0.05). To evaluate the epithelium barrier in the colon mucosa, tight junction (TJs) proteins such as ZO-1, occludin and claudin-1 were detected (Figures 3D,E). The data showed that TJs protein levels were increased in both the cecropin A and the gentamicin groups, compared to the DSS group (*P* < 0.01). The abundance of occludin and claudin-1 proteins was also greater in the cecropin A group than in the gentamicin group (*P* < 0.05). The data suggest that both cecropin A and gentamicin could effectively relieved epithelium disruption in the ileum and colon. In contrast, cecropin A had a better effect on the recovery of the colon mucosa both in morphology and tight junction protein expression.

#### The Suppressive Effect of Cecropin A and Gentamicin on Proinflammatory Cytokines, MAPK and NF-κB Downstream Protein Phosphorylation in Colon Tissue

**FIGURE 4:**
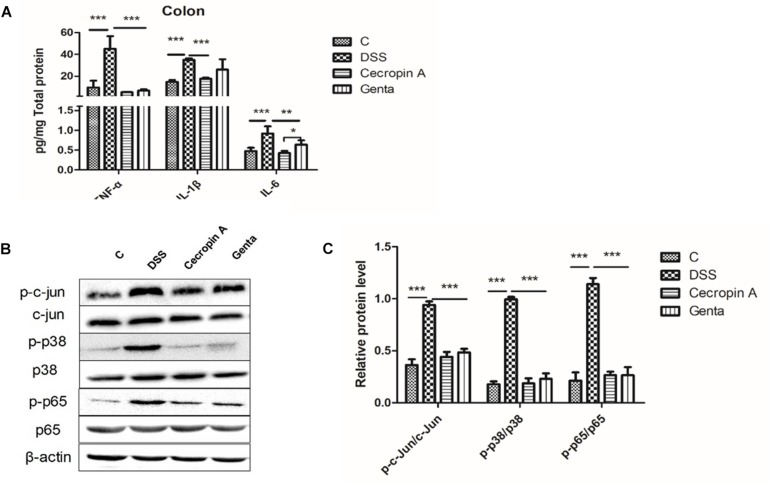
Abundances of pro-inflammatory cytokines, and phosphorylated proteins in the MAPK and NF-κB signaling pathways in the control, DSS, cecropin A and gentamicin groups in mice. **(A)** abundances of TNF-α, IL-1β, and IL-6, *n* = 8; **(B,C)** the phosphorylation levels of p38, c-jun, and NF-κB p65 analyzed by Western blotting, *n* = 3. C: the control group; Genta: the mice were pretreated by 2.5% DSS for 5 days and then received intraperitoneal injection of 5 mg/kg body weight gentamicin. Cecropin A: the mice were pretreated by 2.5% DSS for 5 days and then received intraperitoneal injection of 15 mg/kg body weight cecropin A. DSS: the mice were treated by 2.5% DSS. The control group and DSS group received intraperitoneal injection of saline. The data are mean ± SEM and analyzed by one-way ANOVA. ^*^P < 0.05, ^∗∗^*P* < 0.01, and ^∗∗∗^*P* < 0.001.

TNF-α, IL-1β, and IL-6 levels were readily detected in colon tissues (Figure 4A). DSS treatment induced an increase in TNF-α, IL- 1β, and IL-6 levels. However, cecropin A and gentamicin decreased these three cytokines in comparison with the DSS group. In contrast, the levels of IL-6 in the gentamicin group were higher than those in the cecropin A group (*P* < 0.05). To explore the inhibitory mechanism of cytokines, key proteins (p65 and phosphorylated p65) in the NF-κB pathway and c-jun, p38 in the MAPK pathway were detected (Figures 4B,C). NF-κB p65, c-Jun and p38 phosphorylation levels were upregulated after DSS treatment, however, their levels were decreased in the cecropin A and gentamicin groups (*P* < 0.05).

### Effect of Cecropin A and Gentamicin on Diversity, Richness, and Composition of Bacterial Communities in IBD Mice

The microbiota in cecum contents was analyzed by sequencing the bacterial 16S rRNA V3 + V4 region. A total of 4466203 clean sequences was obtained with an average of 135,339 sequences per sample, with 143,661, 121,409, 126,711, and 147,871 clean sequences in samples from the control, DSS, cecropin A and gentamicin groups, respectively. Based on the 97% similarity, all of the clean reads were clustered to OTUs. The curves of the OTU rank abundance, rarefaction (Supplementary Figures S6A,B), observed species (OS), indexes of Shannon, Simpson, Chao1, and ACE were calculated (Table 1). In our analysis, 522, 439, 501, and 472 species were annotated in those groups, respectively. Rarefaction curves showed that the selected sequences were sufficient to determine most bacterial diversity (Supplementary Figure S6). In IBD- and gentamicin-treated mice, the observed species were significantly decreased when compared to the cecropin A group. The diversity (Shannon) and richness (ACE) indices showed that both cecropin A and gentamicin increased the diversity and richness of the colonic microbiota (*P* < 0.05).

**TABLE 1:** Sequencing data and the alpha diversity in each group of mice.

**Items**	**C**	**DSS**	**Cecropin A**	**Genta**	**SEM**
OS	522^a^	439^b^	501^a^	472^ab^	10
Shannon	6.2073^a^	5.2736^b^	5.8118^a^	5.8738^a^	0.10
Simpson	0.9691^a^	0.9255^b^	0.9605^a^	0.9495^ab^	0.05
Chao1	508.51^a^	459.98^b^	507.17^a^	490.37^ab^	10.89
ACE	539.18^a^	458.83^c^	519.22^a^	490.51^b^	10.55

*Control: the control group (n = 9); Gentamicin: the mice was pretreated by using 2.5% DSS for 5 days and then intraperitoneal inject 5 mg/kg body weight gentamicin (n = 8). Cecropin A: the mice were pretreated by using 2.5% DSS for 5 days and then received intraperitoneal injection of 15 mg/kg body weight cecropin A (n = 9). DSS: The mice treated by 2.5% DSS (n = 7). The control group and DSS group received intraperitoneal injection of saline. The data were shown as mean ± SEM and when significant main effects or interactive effects were observed, the means were compared using the least significant difference (LSD) method. P < 0.05 were taken to indicate statistical significance between groups. Means with different letters “a” (the groups with highest level), “b” (the middle level”) and “c” (the lowest level)” are significantly different. OS, observed species; ACE, abundance-based coverage estimator.*

To better understand the differences in microbial richness or diversity among groups, the overlap was illustrated by using the Venn diagram (Supplementary Figure S7A). The data showed that there were 1,004, 986, 1,056, and 863 OTUs in the control, gentamicin, cecropin A, and DSS groups, respectively. There were 579 common OTUs among the groups, while 155, 89, 114, and 81 unique OTUs in those groups, respectively. By using PCoA, PCA, and NMDS, the plot showed that the groups were clearly separated to different clusters, indicating distinctive microbial communities among the different groups of mice (Supplementary Figures S7B–D).

### Gut Microbiota Composition Differs Between Groups

**FIGURE 5:**
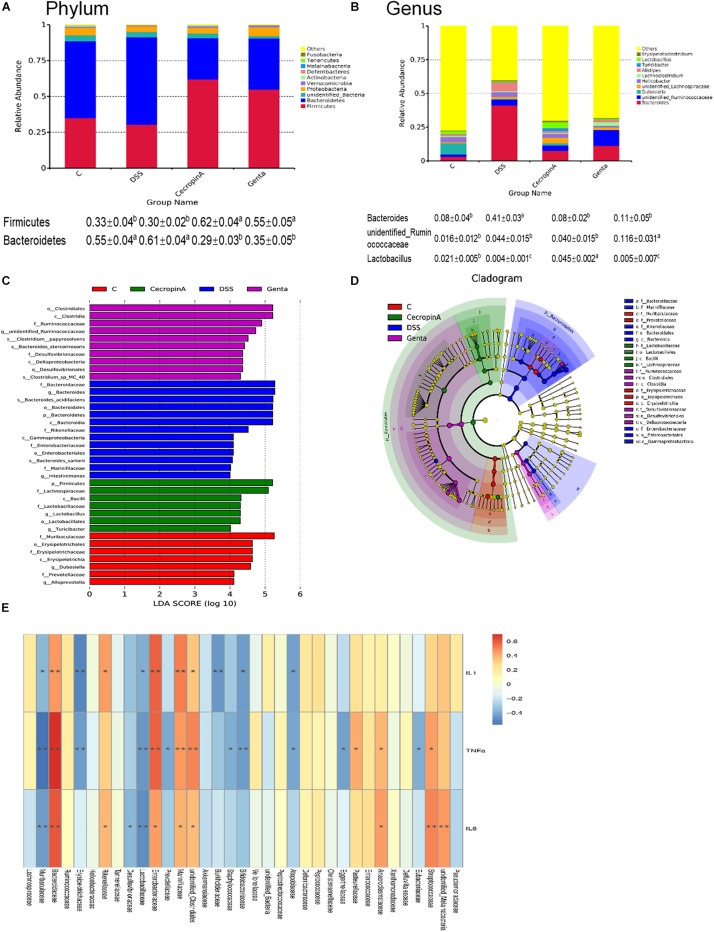
Effects of different treatment on gut microbiota in mice. **(A,B)** the relative abundance of gut microbiota in phylum and genus level. **(C)** LDA score, an LDA score higher than 4 was considered to be an important contributor to the model; **(D)** LEfSe taxonomic cladogram, different colors suggest enrichment of certain taxa in the control group (**C**, red), DSS group (DSS, blue), cecropin A group (green) and gentamicin group (Genta, purple); **(E)** the correlation analysis among gut microbiota and inflammatory cytokines. The size of the circles is based on relative abundance. C: the control group (*n* = 8); DSS: the mice were treated by 2.5% DSS (*n* = 7); cecropin A: the mice were pretreated by 2.5% DSS for 5 days and then were intraperitoneal injected by 15 mg/kg body weight cecropin A (*n* = 9); Genta: the mice were pretreated by 2.5% DSS for 5 days and then were intraperitoneal injected by 5 mg/kg body weight gentamicin (*n* = 8). The control group and DSS group were intraperitoneal injected by physiological saline. The correlation analysis was conducted by using Spearman analysis.

To elucidate the effect of different treatment on microbiota composition, the relative abundance (Figures 5A,B), LEfSe (Figures 5C,D and Table 2) and the correlation of the microbiota with environmental factors (Figure 5E) were measured. Cecropin A and gentamicin significantly increased the relative abundance of *Firmicutes* but decrease the relative abundance of *Bacteroidetes* at the phylum level (*P* < 0.05). At the genus level, both cecropin A and gentamicin decreased the abundance of *Bacteroides*, compared to the DSS group (*P* < 0.05). In addition, cecropin A and gentamicin increased the relative abundance of *Lactobacillus* and *unidentified_Ruminococcaceae*, respectively (*P* < 0.05). To further identify the differences in the key microbiota among treatment groups, the LEfSe analysis was performed (Table 2 and Table S3). The *Erysipelotrichia-Erysipelotrichales-Erysipelotrichaceae-Dubosilla* cells in the control group were more abundant than those in the other groups. DSS increased the relative abundance of *Bacteroidetes-Bacteroidia-Bacteroidales-Bacteroidaceae-Bacteroide* and *Proteobacteria- Gammaproteobacteria-Enterobacteriales-Enterobacteriaceae.* In the cecropin A group, the abundances of *Firmicutes-Bacilli-Lactobacillales-Lactobacillaceae-Lactobacillus* were significantly increased. However, in the gentamicin group, *Clostridia-Clostridiales-Ruminococcaceae* and *Deltaproteobacteria Desul-fovibrionales-Desulfovibrionaceae* were increased (Table 2). Furthermore, we found that *Bacteroidaceae, Enterobacteriaceae, Streptococcaceae*, *Marinifilaceae*, and *unidentified_Clostridiales* were positively but *Lactobacillaceae*, *Dubosilla* and *Eysipelo- trichaceae* were negatively correlated with the levels of TNF-α, IL-1β, and IL-6. Besides, we also found that the higher level of *Alloprevotella*, *Dubosiella* in control group while *Rikenellaceae* and *Alistipes* in DSS treatment group, which may also suggest these flora may also play important roles in IBD treatment (Table S3).

**TABLE 2:** The significant differently species determined based on the LEfSe method.

**Taxon**	**Control**	**DSS**	**Cecropin A**	**Gentamicin**	**SEM**
*Firmicutes| Erysipelotrichia| Erysipelotrichales| Erysipelotrichaceae*	0.0967^a^	0.0212^b^	0.0533^b^	0.015^b^	0.010
*Firmicutes| Bacilli*	0.0273^b^	0.0057^c^	0.047^a^	0.005^c^	0.004
*Firmicutes| Bacilli| Lactobacillales*	0.0219^b^	0.0051^c^	0.0447^a^	0.0047^c^	0.004
*Firmicutes| Bacilli| Lactobacillales| Lactobacillaceae| Lactobacillus*	0.0212^b^	0.0043^c^	0.0447^a^	0.0046^c^	0.004
*Bacteroidetes| Bacteroidia*	0.5466^a^	0.6094^a^	0.2884^b^	0.3542^b^	0.030
*Bacteroidetes| Bacteroidia| Bacteroidales*	0.5463^a^	0.6091^a^	0.2878^b^	0.3538^b^	0.030
*Bacteroidetes| Bacteroidia| Bacteroidales| Bacteroidaceae| Bacteroides*	0.0798^b^	0.4108^a^	0.0754^b^	0.1145^b^	0.029
*Proteobacteria| Gammaproteobacteria*	0.0133^b^	0.027^a^	0.0038^b^	0.0053^b^	0.003
*Proteobacteria| Gammaproteobacteria| Enterobacteriales*	0.0003^b^	0.0235^a^	0.0002^b^	0.0009^b^	0.002
*Proteobacteria| Gammaproteobacteria| Enterobacteriales| Enterobacteriaceae*	0.0003^b^	0.0235^a^	0.0002^b^	0.0009^b^	0.002
*Proteobacteria| Deltaproteobacteria| Desulfovibrionales| Desulfovibrionaceae*	0.029^b^	0.011^b^	0.022^b^	0.0561^a^	0.004
*Firmicutes| Clostridia| Clostridiales*	0.2062^b^	0.276^b^	0.5158^a^	0.5299^a^	0.032
*Firmicutes| Clostridia| Clostridiales| Ruminococcaceae*	0.0554^b^	0.1079^b^	0.1066^b^	0.2141^a^	0.016

*Means with different letters (a–c) are significantly different, P < 0.05.*

## Discussion

The results in this study demonstrated that intraperitoneal injection of cecropin A could alleviate DSS-induced body weight loss, diarrhea, fecal bleeding, while decreasing the levels of pro-inflammatory cytokines in the colon tissues of mice. Accordingly, the 16s rRNA analysis revealed that component and relative abundances of key bacterial species were altered in cecropin A-treated mice, compared with the control group.

IBD is complex in etiology and syndrome, which have been widely studied in recent years. The acute IBD often induces body weight loss, diarrhea, fecal bleeding and even death (Ng et al., 2018). In this study, we constructed a highly lethal IBD model by using 4% DSS in mice. Then different doses of cecropin A were used. Our data showed that 15 mg/kg cecropin A could improve survival rate, body weight gain and gut recovery. Although a low dose (5 mg/kg) of cecropin A had no effect on survival rate, the colon length was increased in the treated mice with IBD, compared to the untreated mice with the disease. We also used gentamicin as the antibiotic control to compare the difference between antibiotic and AMP. Although gentamicin showed similar effects on improving the fecal bleeding and diarrhea, the body weight change was different. Consistent with previous studies, intraperitoneal or rectal administration of AMPs were found to have a therapeutic effect on IBD or diarrhea. For example, AMPs derived from *Allomyrina dichotoma* defensin, CWA derived from *Bungarus fasciatus* and Onc112 could effectively relieve the LPS- or *E⋅coli-*induced intestinal stress and diarrhea in experimental animals (Koyama et al., 2006; Song et al., 2015; Schmidt et al., 2016).

Although the pathogenesis of IBD is complex, the gut microbiota disorder remains one of the most important observations. The species, richness and abundance of gut microbiota were significantly decreased in IBD patients than in healthy people (Zmora et al., 2019). Consistent with previous results, we also found the similar phenomenon in IBD mice. By using the LEfSe analysis, our results also showed that in the IBD mouse model, the relative abundance of *Bacteroides and Enterobacteriaceae* were significantly increased. This indicates that *Bacteroides* and *Enterobacteriaceae* are highly related to IBD. Previous studies showed that *Bacteroides* was closely related to enteritis, appendicitis and other intestinal diseases. For example, *Bacteroides fragilis* could produce enterotoxin, which targets on colonic epithelium cells (Chung et al., 2018) and *Alistipes* was found to be highly correlated with diet and colon health (Fenner et al., 2007; David et al., 2014). *Enterobacteriaceae* is a family of large gram-negative bacteria, in which dozens of species such as *Salmonella*, *Escherichia*, and *Klebsiella* are pathogenic. In the IBD, the relative abundance of harmful flora is generally increased to cause inflammatory damage to the gut epithelium barrier (Ciccone et al., 2015). For example, LPS, a kind of endotoxin produced by the gram-negative bacteria cell wall, could activate the inflammatory signaling pathways such as NF-κB and MAPK. Under this condition, pro-inflammatory cytokines are released to initiate immune response. However, cytokine overexpression may induce autophagy and apoptosis of cells (Jia et al., 2006). On the other hand, the cytokines also accelerate intestinal barrier damage and invasion of pathogens. Our data showed that, in the cecropin A and gentamicin groups, the levels of TNF-α, IL-1β, and IL-6 were significantly lower than those in the DSS group. Consistent with this result, we found that *Bacteroides* and *Enterobacteriaceae*, which are highly correlated with the release of inflammatory cytokines, were decreased to a level similar to that in the control group. Phosphorylation of NF-κB (p65), P38, and c-Jun was also decreased compared to the DSS group. Besides, the intestinal mucosa and TJs protein levels were significantly recovered in the cecropin A and gentamicin groups. The proliferation, adhesion, invasion and damage to gut epithelium are the reasons of IBD. Inflammatory factors are an important part of intestinal immunity in mice. Some inflammatory factors such as TNF-α play an important role in activating the immune system in the early stage of IBD for resistance or defense against the invasion of harmful bacteria. However, the secretion of proinflammatory factors will lead to the damage of intestinal barrier and other negative effects, which will further aggravate the diseases (Neurath, 2014). Our study shows that the abundance of some harmful intestinal flora is highly correlated with the secretion level of proinflammatory factors and the severity of disease in mice. This is not only helpful for researchers to deepen the understanding and application of DSS-induced mouse enteritis model, but also benefits in discovering new drugs targeting specific pathogens or inhibiting the secretion of specific validation factors, thus promoting the research of antibiotic substitutes.

Although both cecropin A and gentamicin can relieve DSS-induced IBD through elimination of enteritis-related harmful bacteria, there are some differences in their effects on the gut microbiota, which may lead to different degrees of recovery from intestinal injury. Our results showed that cecropin A could increase *Lactobacillus* compared to the other groups. *Lactobacillus* is a class of beneficial bacteria for gastrointestinal health. First of all, *Lactobacillus* could maintain intestinal health through interaction with the immune system. *Lactobacillus reuteri* could suppress TNF-α expression through inhibiting the activation of MAP kinase-regulated c-Jun (Lin et al., 2008). In addition, γ-aminobutyric acid, one of the metabolites of *Lactobacillus* mediates interleukin-17 expression and relieving diarrhea in piglets and mice (Ren et al., 2016). Secondly, *Lactobacillus* could compete with pathogenic bacteria and decrease the abundance. *Lactobacillus gasseri LA39* could release a peptide called gassericin A, thereby preventing diarrhea in piglets (Hu et al., 2018). Third, part of *lactobacillus* has protective effects against intestinal epithelial dysfunction. For example, oral administration of *Lactobacillus plantarum* or *Lactobacillus rhamnosus GG* could increase intestinal barrier function and TJ protein expression (Yang et al., 2014; Chen et al., 2016). In consistent with the previous studies, our H&E staining showed that cecropin A had a better effect on the intestinal and colon mucosa recovery compared to the gentamicin group. Besides, the protein levels of TJs, which play important roles in epithelium barrier and preventing the invasion of pathogenic microorganisms, were also detected in the colon of mice. Specifically, compared to gentamicin, cecropin A had no effect on ZO-1, but increased the protein levels of occludin and claudin-1. In the gentamicin group, the relative abundances of *Ruminococcaceae* and *Desulfovibrionaceae* were increased. As was shown in the previous studies with the human colon, digestion of resistant starch depends on *Ruminococcaceae* (Ze et al., 2012). *Desulfovibrionaceae*, which is a family of gram-negative sulfate-reducing intestinal bacteria, induce the reduction of sulfate. Although the relationship among *Ruminococcaceae*, *Desulfovibrionaceae* and IBD remains unclear, researchers have reported that the abundance of the two families are increased in the feces of IBD patients (Berry and Reinisch, 2013). Besides, previous studies showed that hydrogen sulfide, a main metabolite of *Desulfovibrionaceae*, may be a breaker of intestinal mucosal barrier and induces IBD (Ijssennagger et al., 2016). Interestingly, although the two kinds of bacteria are highly correlated with enteritis, we found that they were not increased in the DSS group, compared with the control group. This may be result from differences in species and diet, as well as DSS-induced mouse models and IBD patients.

In conclusion, we found that cecropin A had a therapeutic effect on DSS-induced IBD in mice. Cecropin A and gentamicin showed different effects on their microbiota population. An increase in *Lactobacillus* may be the key factor for better intestinal mucosa recovery after the cecropin A treatment. We also found that gentamicin increased the abundance of *Ruminococcaceae* and *Desulfovibrionaceae*, which may impair intestinal function. This study may provide a new insight into the effects of AMPs on gut microbiota and health.

## Conclusion

This study not only demonstrated the treatment effect of cecropin A on DSS induced IBD, but also demonstrated the similarities and differences compared to gentamicin. Both of cecropin A and gentamicin could decrease the level of floras such as *Bacteroides* and *Enterobacteriaceae* which is positively correlated to IBD. Gentamicin could increase the level of *Desulfovibrionaceae* and *Ruminococcaceae*, while cecropin A could specifically increase the level of *Lactobacillus*, which may benefit for the intestinal epithelium recovery. These results suggest that cecropin A could be a potential substitution of antibiotics, and providing insight in to the differences of the gut microbiota regulation between antimicrobial peptides and antibiotics.

## Data Availability

The datasets generated for this study can be found in the National Center for Biotechnology Information SUB5465416.

## Author Contributions

ZZ and BD designed the study and wrote the manuscript. ZZ, FZ, RC, XN, and ZX performed the experiments. GW revised the manuscript and helped to interpret the results. WR, JD, and YY helped in proofreading of the manuscript.

## Conflict of Interest Statement

The authors declare that the research was conducted in the absence of any commercial or financial relationships that could be construed as a potential conflict of interest.
