# Supplementary material for: Cecropin A Alleviates Inflammation Through Modulating the Gut Microbiota of C57BL/6 Mice With DSS-Induced IBD
Source: Front Microbiol. 2019 Jul 10;10:1595. doi: 10.3389/fmicb.2019.01595 (PMC6635700; doi:10.3389/fmicb.2019.01595)
Supplement: Supplementary file 1 [file Table_1.docx]

**Supporting Information**

**Materials and Methods**

**2.2 Effects of intraperitoneal injection of different doses of cecropin A on mice**

The mice were housed until 6 weeks of age, and then divided randomly into four groups (n=9 per group). The control group mice were intraperitoneally injected with 100 μL physiological saline per mice. The other three groups of mice were intraperitoneally injected with 5 mg/kg, 15mg/kg and 30 mg/kg cecropin A (DgPeptides Co., Ltd., Hangzhou, China) dissolved in 100 μL physiological saline according to the body weight in mice for 5 days. All the mice were weighed every day. Then the mice were sacrificed and the blood samples were centrifuged at 3000 rpm for 20 min to collect the serum. The serum samples were sub-packaged and stored at -80℃ until further analysis. The activity of lactate dehydrogenase (LDH), alkaline phosphatase (AKP) and aspartate aminotransferase (AST) were detected by using assay kits (Nanjing Jiancheng Bioengineering Institute, Nanjing, China). The liver, spleen, distal ileum and colon samples were collected and stored in 4% neutral polyformaldehyde fixative for 48h, and then the tissue was embedded with paraffin for further section and staining.

**Supporting Information Tables**

**Table S1 The disease activity score parameters for IBD**

| Score | Weight loss | Stool Consistency | Bleeding |
| --- | --- | --- | --- |
| 0 | None or gain | Formed | Normal color |
| 1 | 5%-10% loss | Mild-soft | minimal blood in stool |
| 2 | 10%-15% loss | Diarrhea | Mild blood in stool |
| 3 | 15%-20% | watery stool | evidence blood in stool |
| 4 | ＞20% | Gross diarrhea | only blood |

**Table S2. The histologic scoring system**

| Inflammation | Crypt Injury | Ulceration | Score |
| --- | --- | --- | --- |
| No significant inflammation | No injury | No ulceration | 0 |
| Neutrophilic inﬂammation in epithelium or lamina propria | Loss of basal one-third of crypts | 2 or less foci of ulceration | 1 |
| Inﬂammatory cells extending into submucosa | Loss of basal two-thirds of crypts | 3 or 4 foci of ulceration | 2 |
| Transmural inflammation | Loss of full thickness crypts | Diffuse/conﬂuent ulceration | 3 |
|  | Full thickness crypt loss with surface erosion |  | 4 |

**Table S3 The significant differently species determined**

*P* values < 0.05 were taken to indicate statistical significance between groups. Means with different letters “a” (the groups with highest level), “b” (the middle level”) and “c” (the lowest level)”, are significantly different.

|  | Control | DSS | Cecropin A | Gentamicin | SEM |
| --- | --- | --- | --- | --- | --- |
| Bacteria\|Bacteroidetes\|Bacteroidia\|Bacteroidales\|Prevotellaceae\|Alloprevotella | 0.0249^a^ | 0.0002^b^ | 0.0014^b^ | 0.0002^b^ | 0.0024 |
| Bacteria\|Bacteroidetes\|Bacteroidia\|Bacteroidales\|Rikenellaceae | 0.0135^b^ | 0.0796^a^ | 0.0235^b^ | 0.0338^b^ | 0.0664 |
| Bacteria\|Bacteroidetes\|Bacteroidia\|Bacteroidales\|Rikenellaceae\|Alistipes | 0.0112^b^ | 0.0661^a^ | 0.0142^b^ | 0.0172^b^ | 0.0296 |
| Bacteria\|Firmicutes\|Erysipelotrichia\|Erysipelotrichales\|Erysipelotrichaceae\|Dubosiella | 0.0721^a^ | 0.0062^b^ | 0.0151^b^ | 0.0007^b^ | 0.008 |

**Supporting Information Figures**

**A**

**B**


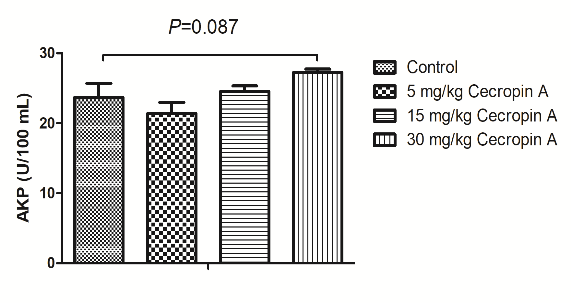

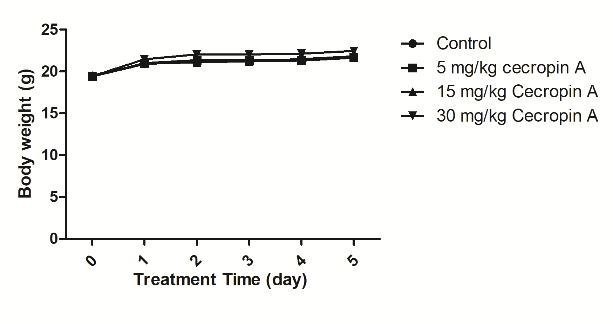

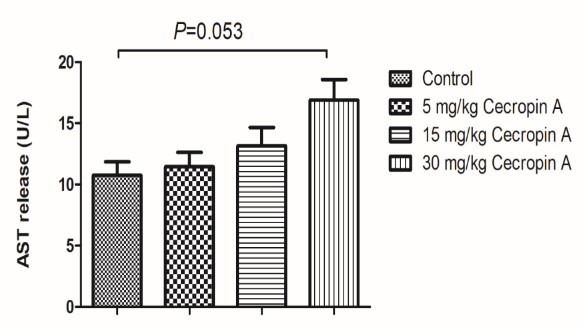

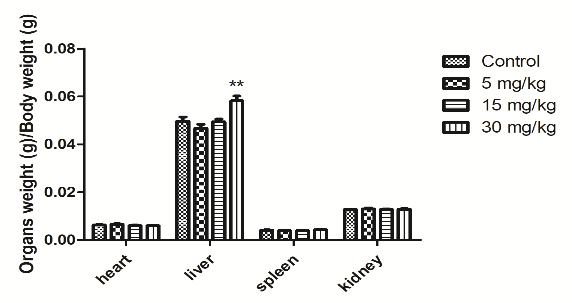


**C**

**D**

**E**


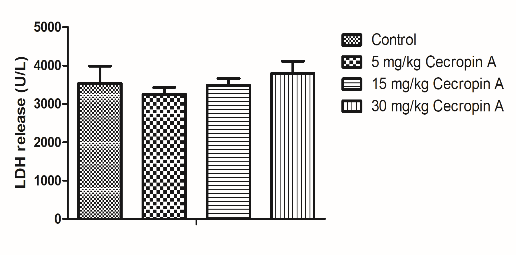


**Figure S1.** Intraperitoneal injection of cecropin A have no effect on body weight and heart, spleen and kidney index but increased the liver index of mice. A) The body weight; B) the organ index; C) AST concentration in serum; D) AKP concentration in serum. E) LDH concentration in serum. The mice received intraperitoneal injection of saline, 5, 15 or 30 mg/kg cecropin A and from day 0 to 5. The data (mean ±SEM) were analyzed by using one-way ANOVA, (n=9). ** *P*< 0.01. The organ index was shown as organ weight to body weight ratio. The data (mean ±SEM) were analysed by using one-way ANOVA, (n=9). ** *P*< 0.01.


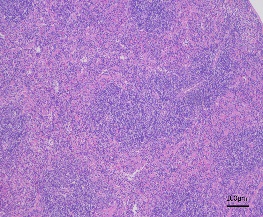

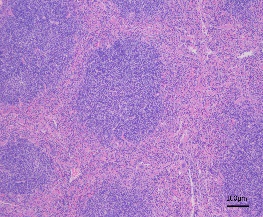

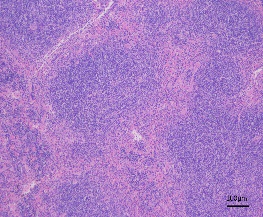

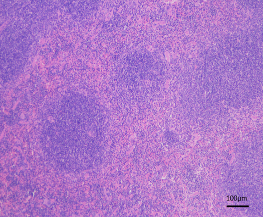


Control

5 mg/kg cecropin A

15 mg/kg cecropin A

30 mg/kg cecropin A

Spleen


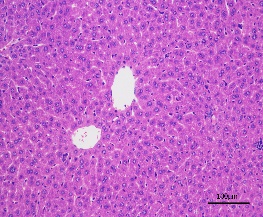

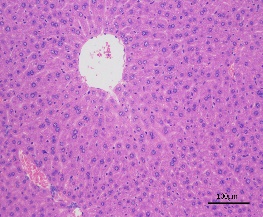

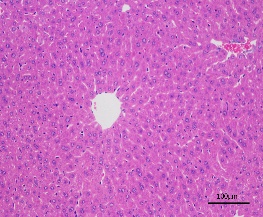

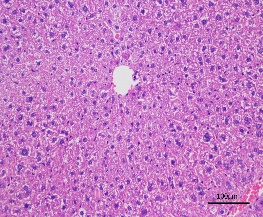


a

b

c

d

Liver

e

f

g

h

**Figure S2.** Intraperitoneal injection of cecropin A have no effects on liver morphology in 5 mg/kg and 15 mg/kg group but changes the liver cell morphology in 30 mg/kg group but have no effect on spleen morphology in all the groups. The representative images of H&E staining in spleen (a-d) and liver (e-h), 200×, n=5.

Control


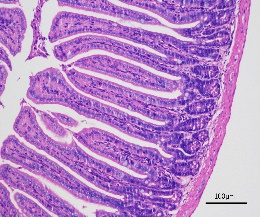

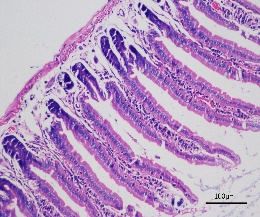

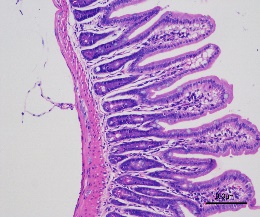


5 mg/kg Cecropin A

15 mg/kg Cecropin A

30 mg/kg Cecropin A


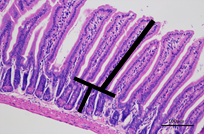

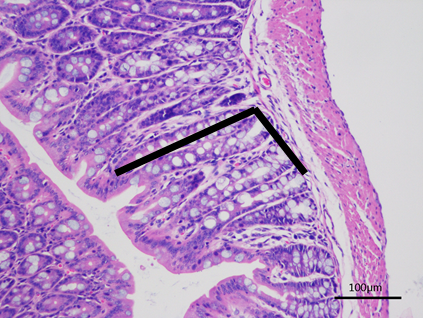

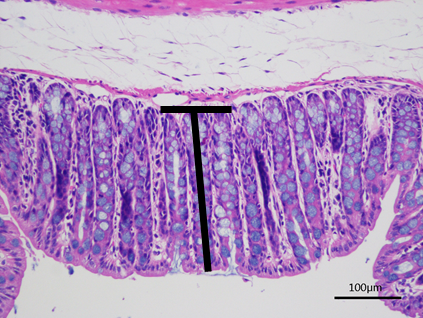

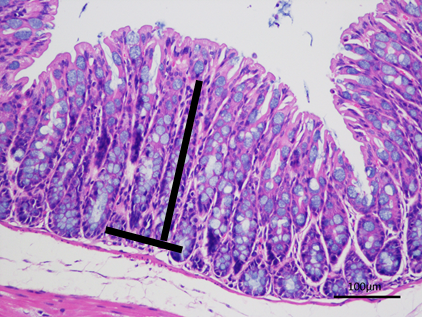

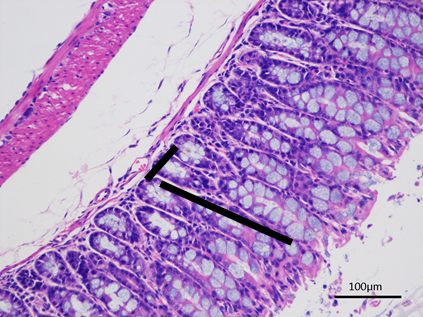


Ileum

Colon


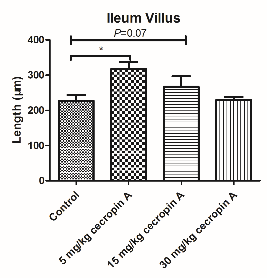

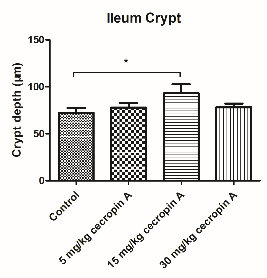

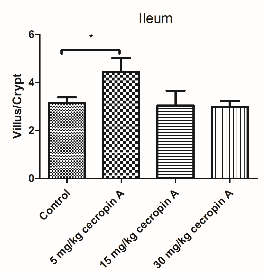

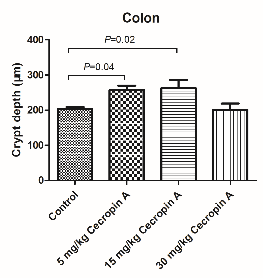


a

b

c

d

e

f

g

h

i

j

k

l

**Figure S3.** Intraperitoneal injection of cecropin A influenced the ileum and colon morphology in DSS-treated mice. The representative images of H&E staining in lieum (a-d) and colon (e-h), 200×, n=5. The villus length and crypt depth were measured as indicated in the image. The statistical analysis of villus length, crypt depth and the ratio of villus to crypt from images also shown (i-l). The data related to villus length and crypt depth were analyzed by one-way ANOVA. The data are shown in mean ± SEM with, **P*<0.05, n = 5.

A


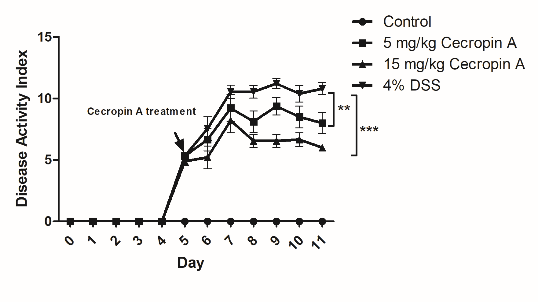

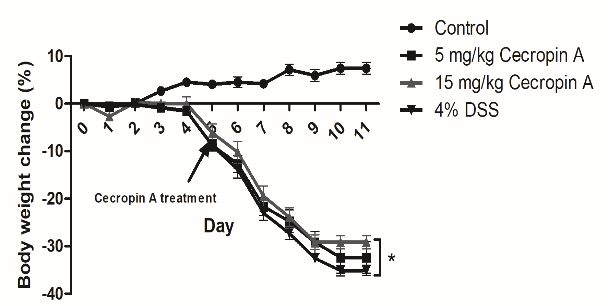

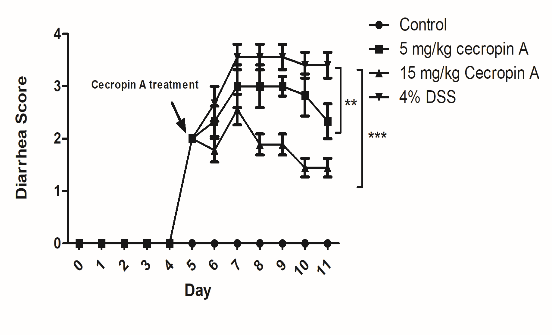


B

C

**Fig. S4** The therapeutic effect of intraperitoneal injection of cecropin A on body weight change, diarrhea score and DAI. A) the body weight; B) diarrhea score; C) DAI score. The diarrhea score and DAI measured by one-way ANOVA, the data are shown as mean ± SEM with, **P*<0.05, ** *P*<0.01, ****P*<0.001. Because of the survival rate was changed every day, except for the control group (n=9), the n number were changed from 4-9 in 5 mg/kg, 15 mg/kg cecropin A or 4% DSS group.


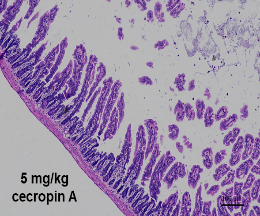

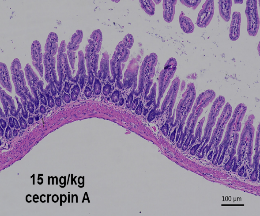

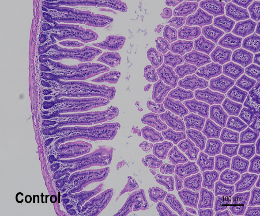

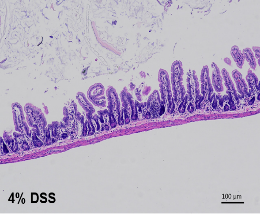


Ileum

a

b

c

d

**Fig. S5** The therapeutic effect of intraperitoneal injection of cecropin A on the ileum morphology in DSS-treated mice. The representative images of H&E staining in ileum (a-d), 100×, n=5.

A


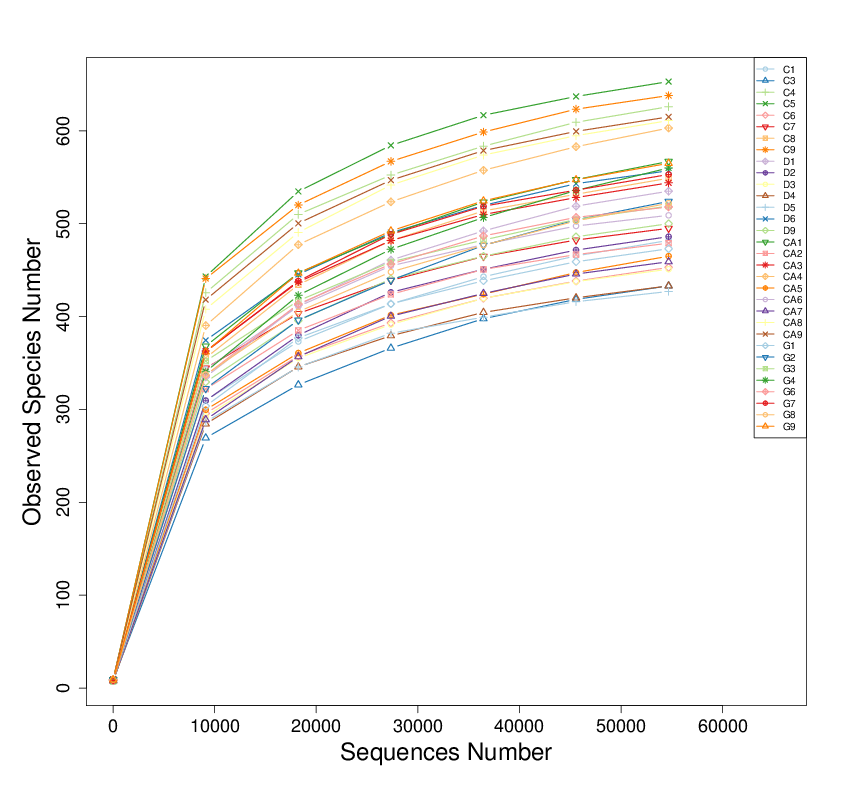

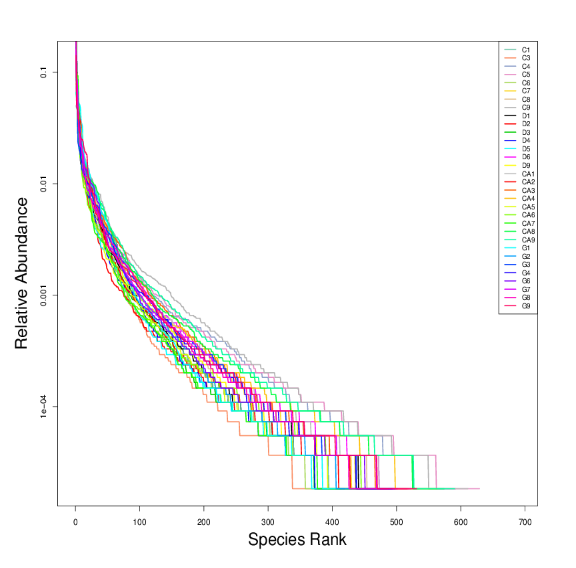


B

**Fig. S6** The Rarefaction curve (A) and rank abundance curve (B). C: control group; D: DSS group, the mouse treated by 2.5% DSS for 5 days; CA: cecropin A group, the mouse was pretreated by using 2.5% DSS for 5 days and then intraperitoneal inject 15 mg/kg body weight cecropin A; G: gentamicin group, the mouse was pretreated by using 2.5% DSS for 5 days and then intraperitoneal inject 5 mg/kg body weight gentamicin. The control group and DSS group were intraperitoneal injected physiological saline.


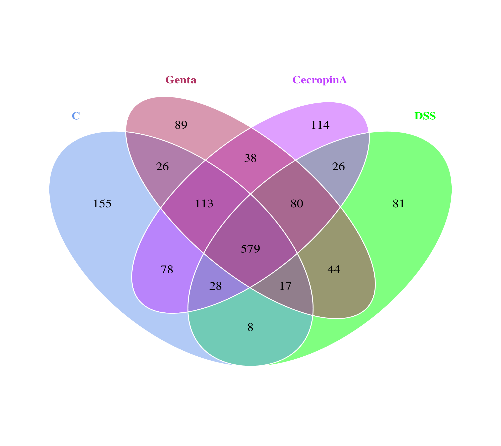


A

B

C

D


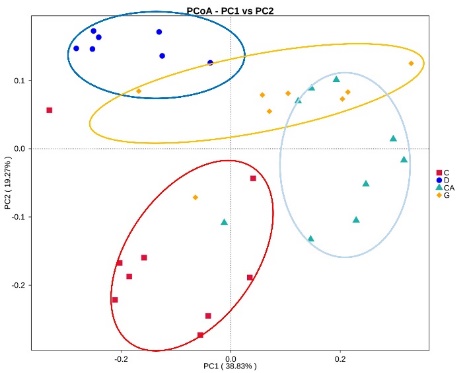

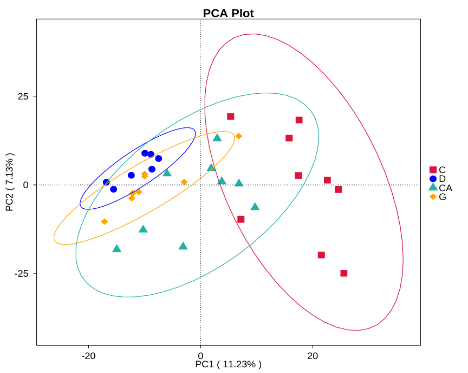

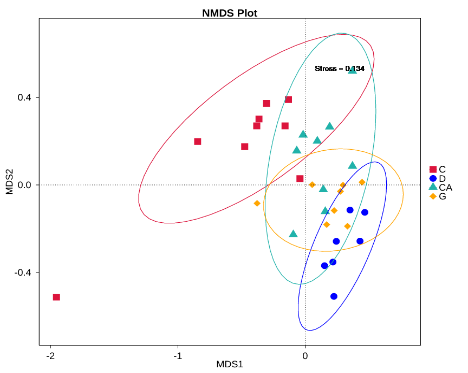


**Figure S7.** Effects on the gut microbiota among the groups. Control group (C, red), DSS group (D, blue), cecropin A group (CA, green) and gentamicin group (G, purple) using unsupervised multivariate statistical methods. (A) Venn diagram; (B) 2D plot of principal coordinate analysis (PCoA); (C) 2D plot of principal component analysis (PCA); (D) 2D plot of non-metric multi-dimensional scaling (NMDS). C: the control group (n=8); G: the mice were pretreated by 2.5% DSS for 5 days and then were intraperitoneal injected by 5 mg/kg body weight gentamicin (n=8). CA: the mice were pretreated by 2.5% DSS for 5 days and then were intraperitoneal injected by 15 mg/kg body weight cecropin A (n=9). DSS: the mice were treated by 2.5% DSS (n=7). The control group and DSS group were intraperitoneal injected by physiological saline.

Graphic Abstract


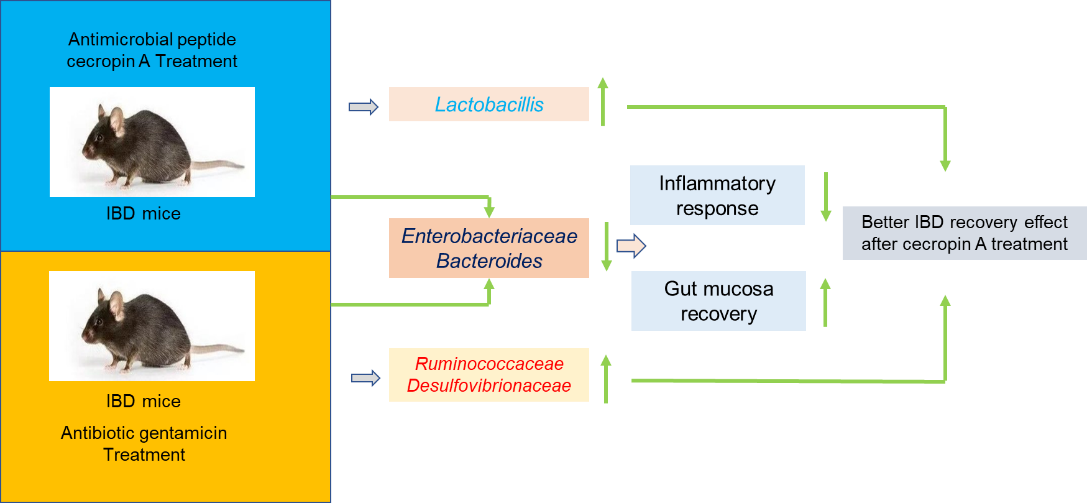


**References:**

[1] F. Zhou, T. Hamza, A. S. Fleur, Y. Zhang, H. Yu, K. Chen, J. E. Heath, Y. Chen, H. Huang, H. Feng, *Inflamm Bowel Dis* **2018**, *24*, 573-582.

[2] L. De Fazio, E. Spisni, E. Cavazza, A. Strillacci, M. Candela, M. Centanni, C. Ricci, F. Rizzello, M. Campieri, M. C. Valerii, *Front Pharmacol* **2016**, *7*, 38.
